# Supplementary material for: Selective photoelectrochemical oxidation of glycerol to high value-added dihydroxyacetone
Source: Nat Commun. 2019 Apr 16;10:1779. doi: 10.1038/s41467-019-09788-5 (PMC6467901; doi:10.1038/s41467-019-09788-5)
Supplement: Supplementary file 1 — Supplementary Information [file 41467_2019_9788_MOESM1_ESM.pdf]

Supplementary Information

Selective Photoelectrochemical Oxidation of Glycerol to High Value-Added Dihydroxyacetone

Liu *et al.*

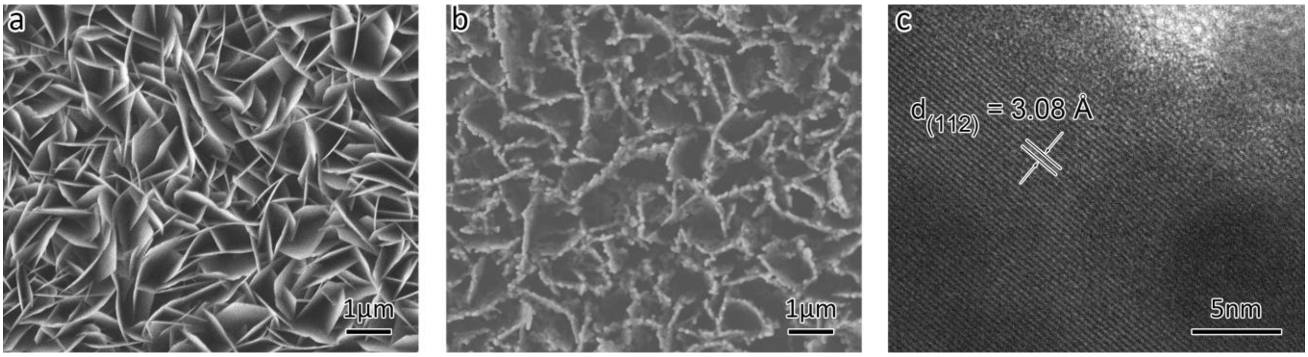

Supplementary Figure 1 | Electron microscope images of BiOI and $BiVO_4$. **a, b,** Top-view SEM images of BiOI nanoflake arrays and $BiVO_4$ nanoporous arrays. **c,** HRTEM image of $BiVO_4$ nanoporous arrays.

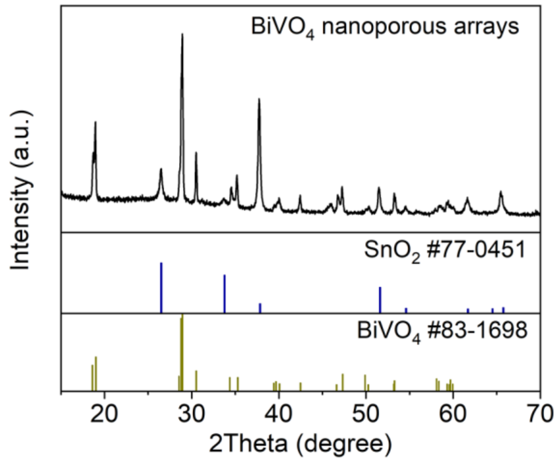

Supplementary Figure 2 | X-ray diffraction pattern of $BiVO_4$ nanoporous arrays fabricated on FTO glass.

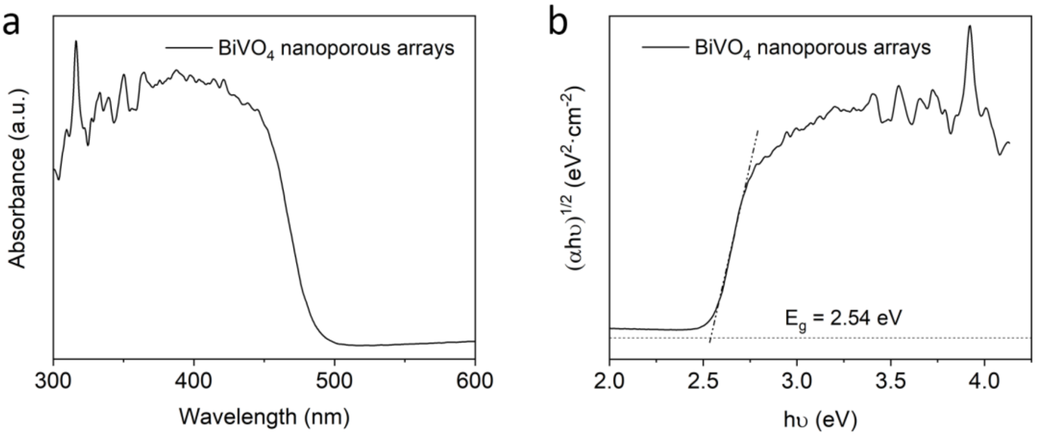

Supplementary Figure 3 | Ultraviolet–visible diffuse reflectance spectrum and bandgap of $BiVO_4$ nanoporous arrays.

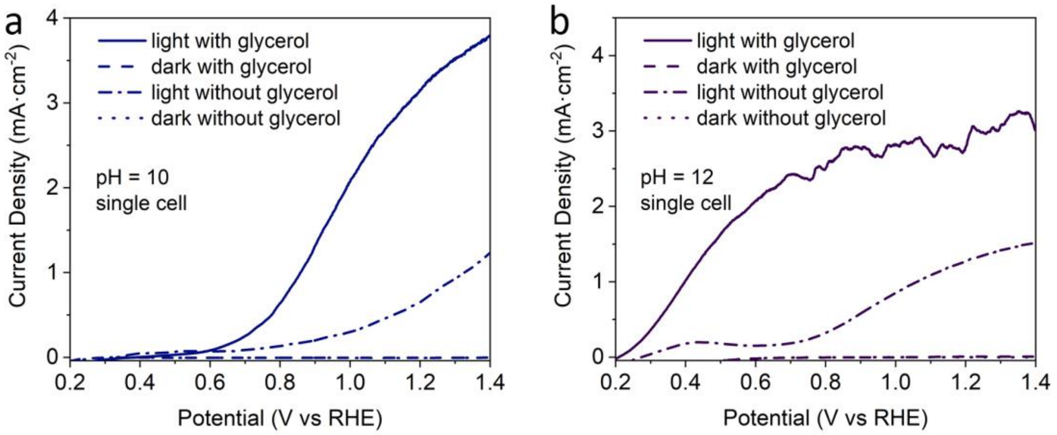

Supplementary Figure 4 | Photoelectrochemical performances in alkaline electrolyte. **a, b,** Current density-potential profiles of $BiVO_4$ photoanode measured in 0.5 M Na_2SO_4 at pH = 10 and pH = 12 in a single cell under dark and AM 1.5G, 100 mW cm^{-2} illumination.

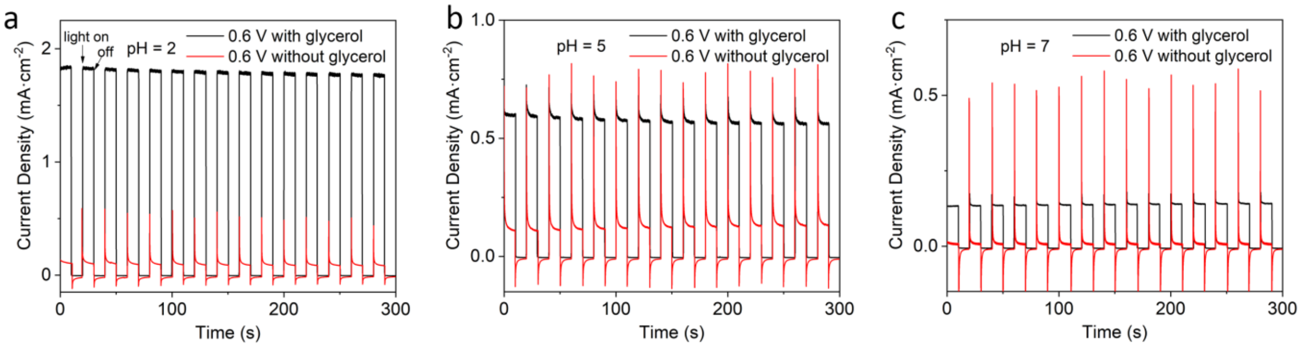

Supplementary Figure 5 | Chopped photocurrent density-time profiles. Chopped photocurrent density-time profiles of $BiVO_4$ at 0.6 V vs. RHE in 0.5 M Na_2SO_4 at various pH with and without glycerol. Source data are provided as a Source Data file.

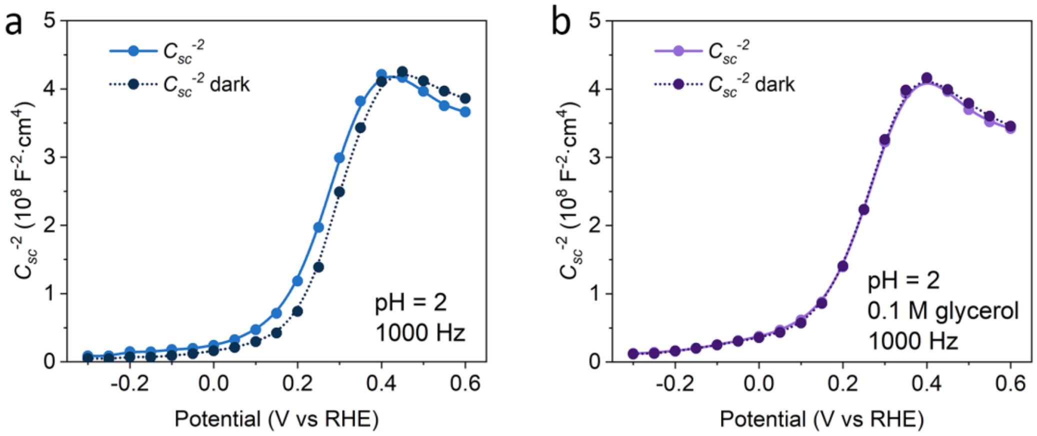

Supplementary Figure 6 | Mott-Schottky plots. Mott-Schottky plots of the $BiVO_4$ photoanode measured in 0.5 M Na_2SO_4 at pH = 2 under dark and AM 1.5G illumination without and with presence of glycerol.

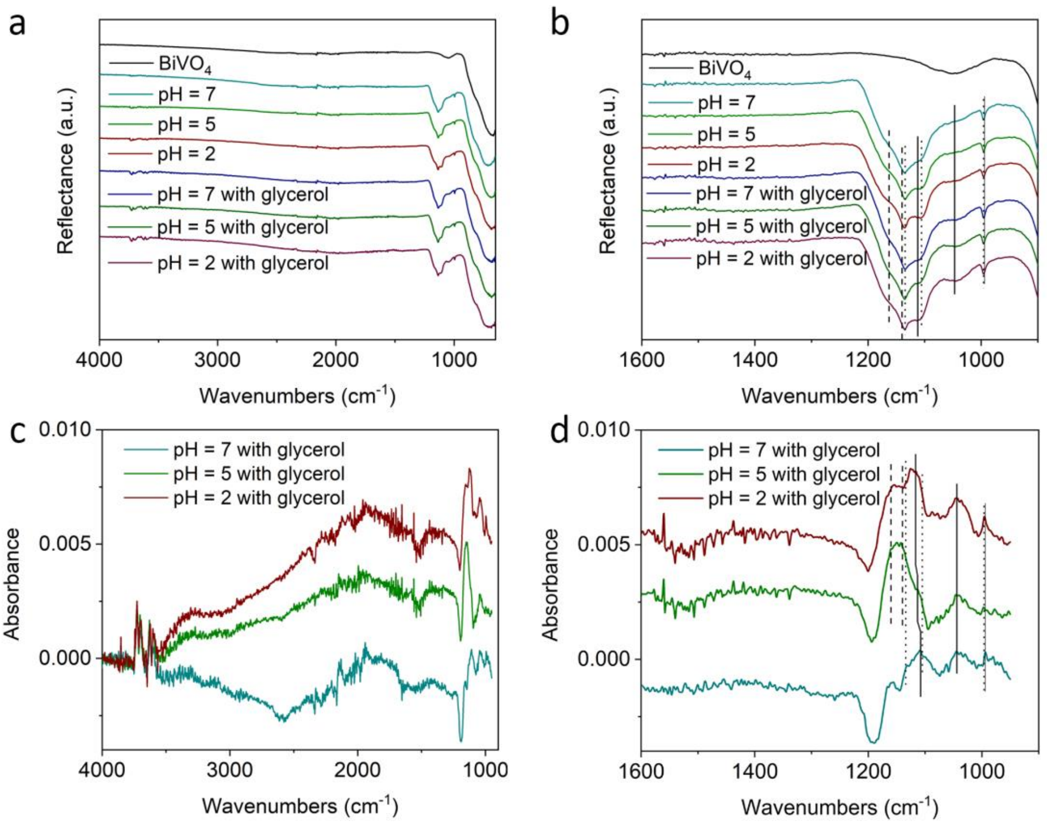

Supplementary Figure 7 | ATR-FTIR spectra of $BiVO_4$ photoanode. a, b, ATR-FTIR reflectance spectra of $BiVO_4$ photoanode treated with solution at pH = 2, 5 and 7. **c, d,** ATR-FTIR absorbance spectra of $BiVO_4$ photoanode treated with glycerol solution at pH = 2, 5 and 7. Absorbance spectra were calculated from the normalized ATR reflectance spectra of $BiVO_4$ samples. Characteristic IR absorbance peaks of glycerol, Na_2SO_4 and H_2SO_4 are labeled with solid line, dotted line and dashed line, respectively.

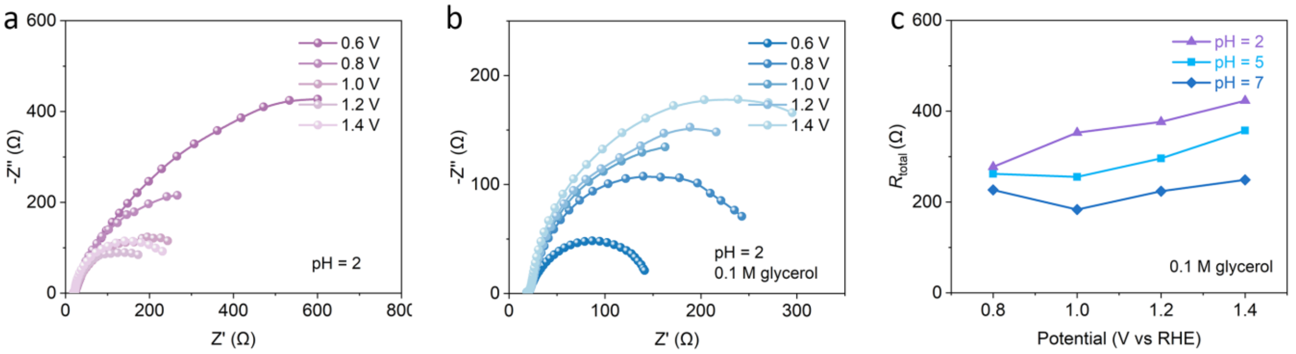

Supplementary Figure 8 | Electrochemical impedance spectroscopy of $BiVO_4$ photoanode. a, b, The Nyquist plots of the EIS data measured under different potentials with AM 1.5G, 100 mW cm^{-2} illumination without and with presence of glycerol. **c,** R_{total} calculated from EIS data at various pH with glycerol under illumination.

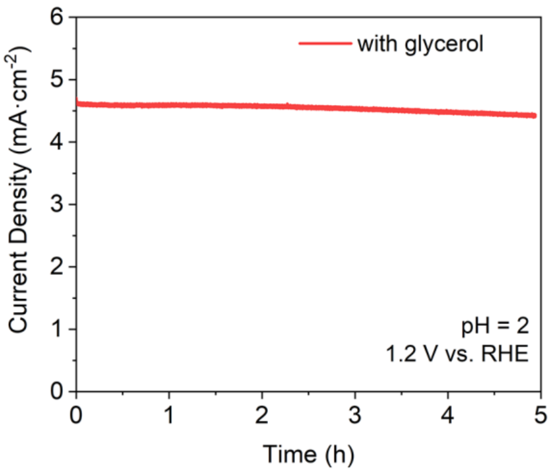

Supplementary Figure 9 | Long time photoelectrochemical stability. Long time stability of $BiVO_4$ photoanode at 1.2 V vs. RHE in 0.5 M Na_2SO_4 at pH = 2 with 1 M glycerol under AM 1.5G, 100 mW cm^{-2} illumination.

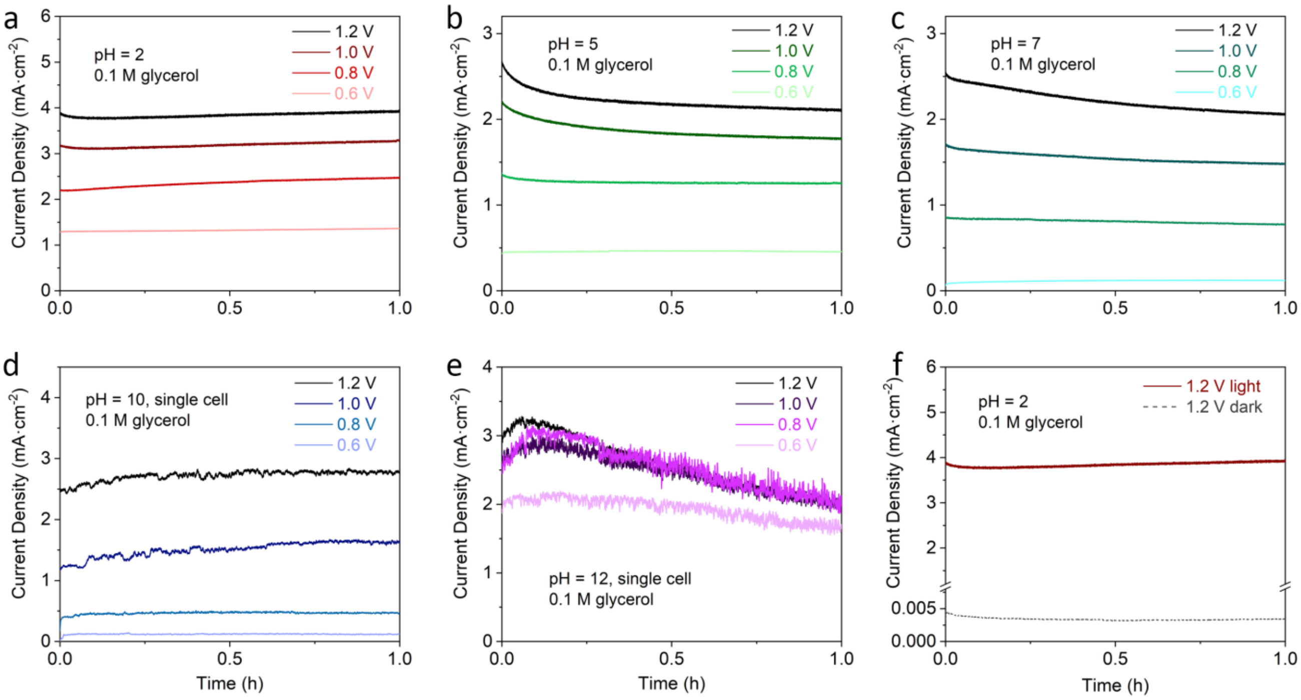

Supplementary Figure 10 | Current density-time curves of $BiVO_4$ photoanode. a, b, c, Photocurrent density-time curves of $BiVO_4$ photoanode measured in 0.5 M Na_2SO_4 at pH = 2, 5 and 7 in an H-type cell at different applied biases under AM 1.5G, 100 mW cm^{-2} illumination. Source data are provided as a Source Data file. **d, e,** Photocurrent density-time curves of $BiVO_4$ photoanode measured in 0.5 M Na_2SO_4 at pH = 10 and 12 in a single cell at different applied biases under AM 1.5G, 100 mW cm^{-2} illumination. **f,** Current density-time curves of $BiVO_4$ photoanode measured in 0.5 M Na_2SO_4 at pH = 2 in an H-type cell at 1.2 V vs. RHE under dark and AM 1.5G, 100 mW cm^{-2} illumination.

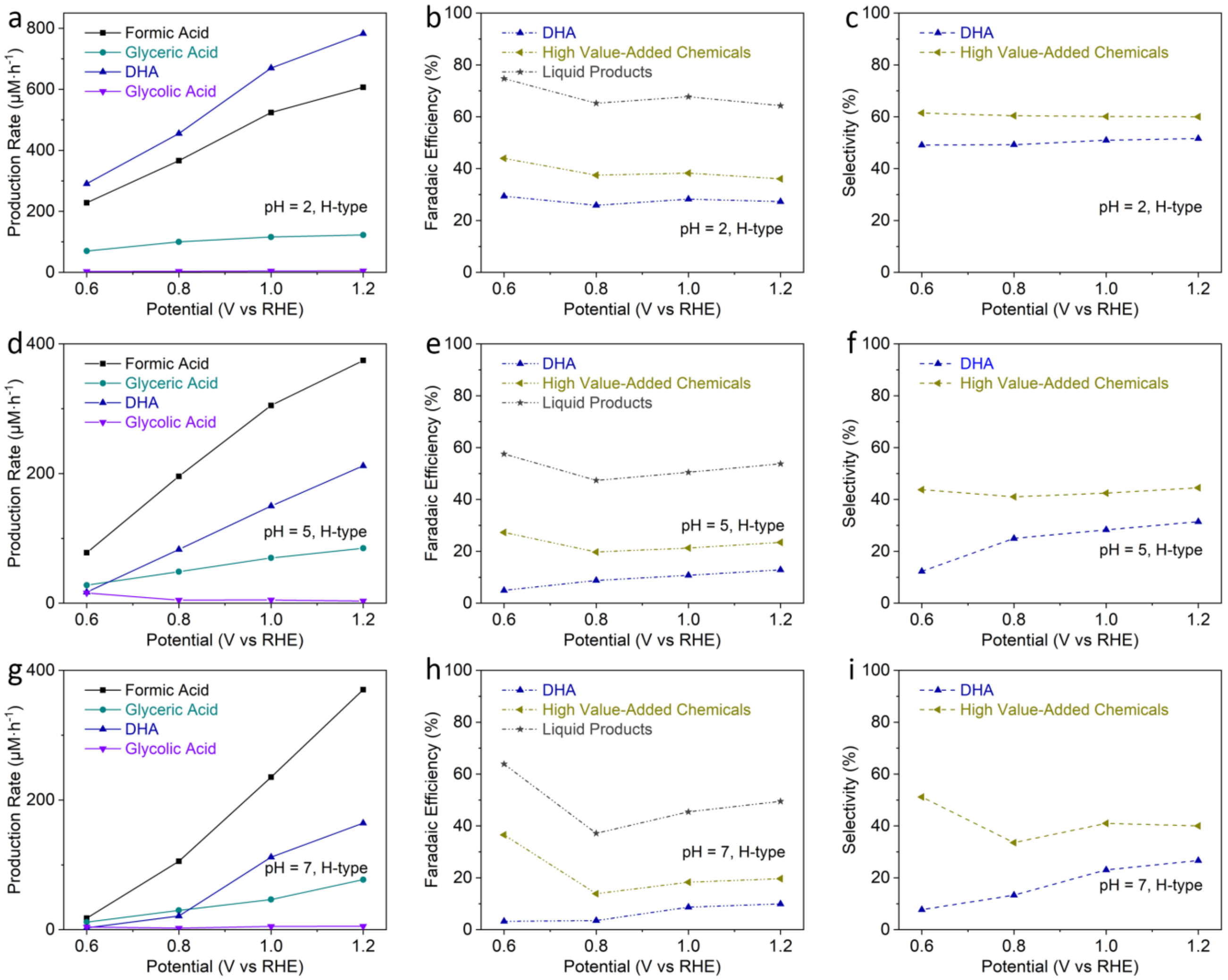

Supplementary Figure 11 | Production rate, faradaic efficiency and selectivity of the main products. Obtained in 0.5 M Na_2SO_4 at various pH in an H-type cell (volume 25 mL) under AM 1.5G, 100 mW cm^{-2} illumination.

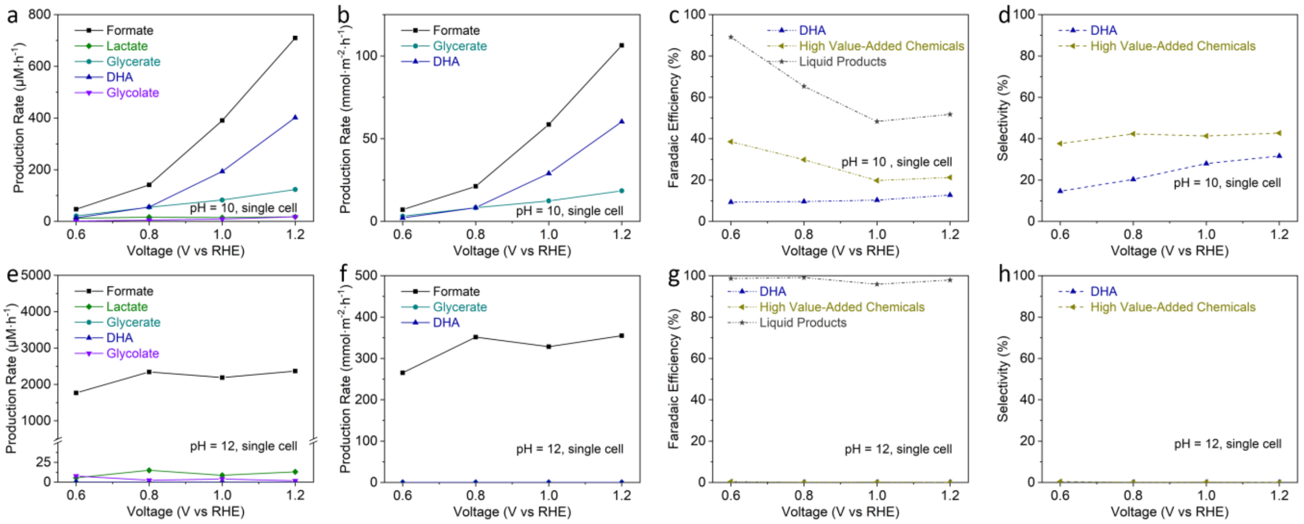

Supplementary Figure 12 | Production rate, faradaic efficiency and selectivity of the main products in alkaline electrolyte. Obtained in 0.5 M Na_2SO_4 at various pH in a single cell (volume 15 mL) under AM 1.5G, 100 mW cm^{-2} illumination.

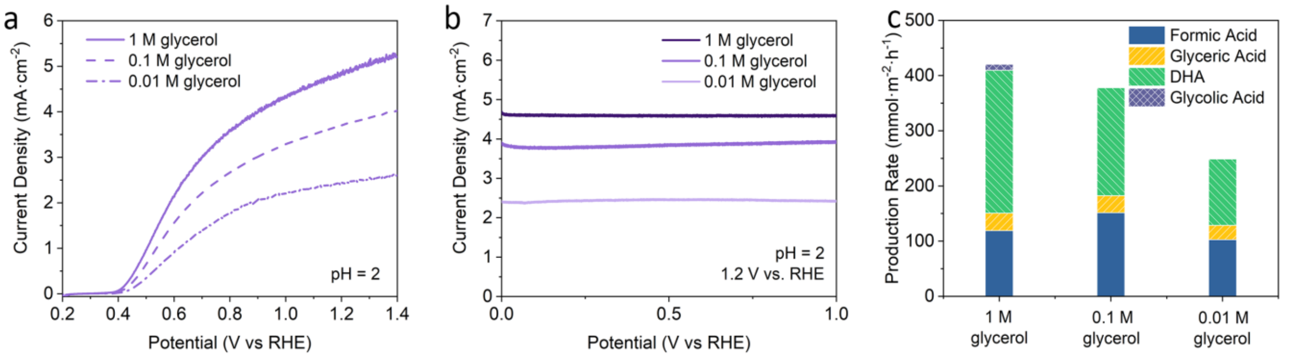

Supplementary Figure 13 | Photoelectrochemical characterization of $BiVO_4$ photoanode with glycerol of different concentrations. a, Photocurrent density-potential profiles of porous $BiVO_4$ nanoarray photoanode measured at pH = 2 and 1.2 V vs. RHE under AM 1.5G, 100 mW cm^{-2} illumination with 1 M, 0.1 M and 0.01 M glycerol. **b,** Current density-time curves of $BiVO_4$ photoanode measured at pH = 2 and 1.2 V vs. RHE under AM 1.5G, 100 mW cm^{-2} illumination with 1 M, 0.1 M and 0.01 M glycerol. **c,** Photoelectrocatalytic production rate of oxidation products at pH = 2 and 1.2 V vs. RHE with glycerol of different concentration.

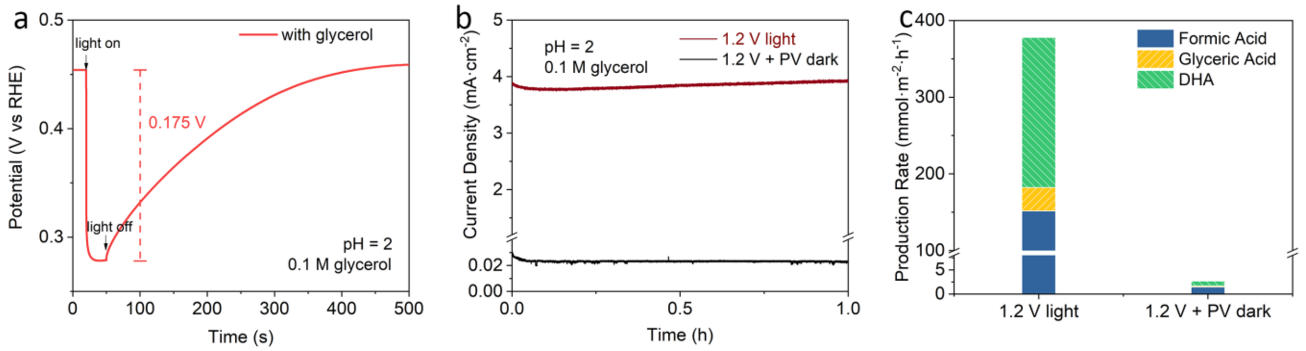

Supplementary Figure 14 | Electrochemical characterization of $BiVO_4$ photoanode with and without light illumination. a, Transient OCVD profile of porous $BiVO_4$ nanoarray photoanode in 0.5 M Na_2SO_4 at pH = 2 with 0.1 M glycerol. **b,** Current density-time curves of $BiVO_4$ photoanode measured in 0.5 M Na_2SO_4 at pH = 2 and 1.2 V vs. RHE under illumination or 1.375 V vs. RHE in dark, respectively. **c,** Production rate of liquid oxidation products at pH = 2 and 1.2 V vs. RHE under illumination or 1.375 V vs. RHE in dark, respectively.

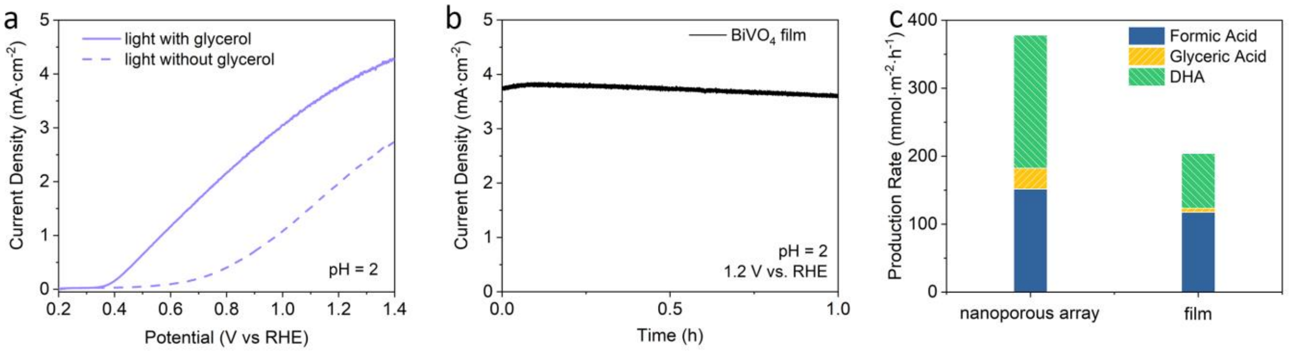

Supplementary Figure 15 | Photoelectrochemical characterization of $BiVO_4$ film photoanode. a, Photocurrent density-potential profiles of $BiVO_4$ film photoanode measured at pH = 2 and 1.2 V vs. RHE under AM 1.5G, 100 mW cm^{-2} illumination with and without 0.1 M glycerol. **b,** Photocurrent density-time curve of $BiVO_4$ film photoanode measured at pH = 2 and 1.2 V vs. RHE under AM 1.5G, 100 mW cm^{-2} illumination with 0.1 M glycerol. **c,** Photoelectrocatalytic production rate of oxidation products at pH = 2 and 1.2 V vs. RHE produced by $BiVO_4$ film photoanode with 0.1 M glycerol.

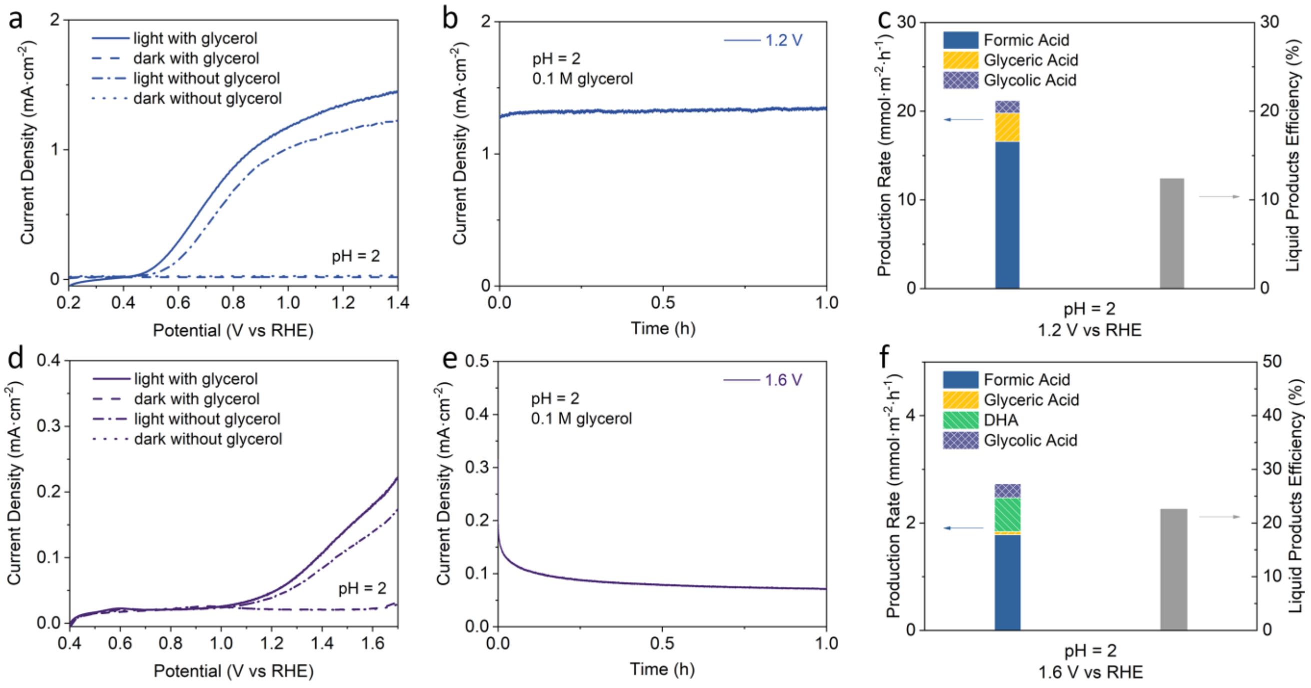

Supplementary Figure 16 | Photoelectrochemical characterization of TiO_2 and Fe_2O_3 photoanode. a, d, Current density-potential profiles of TiO_2 nanowires and Fe_2O_3 nanoporous photoanode measured in 0.5 M Na_2SO_4 at pH = 2 under dark and AM 1.5G, 100 mW cm^{-2} illumination. **b, e,** Photocurrent density-time curves of TiO_2 nanowires and Fe_2O_3 nanoporous photoanode measured in 0.5 M Na_2SO_4 at pH = 2 under AM 1.5G, 100 mW cm^{-2} illumination. **c, f,** Production rate and efficiency of the main liquid products obtained by TiO_2 nanowires and Fe_2O_3 nanoporous photoanode in 0.5 M Na_2SO_4 at pH = 2 under AM 1.5G, 100 mW cm^{-2} illumination.

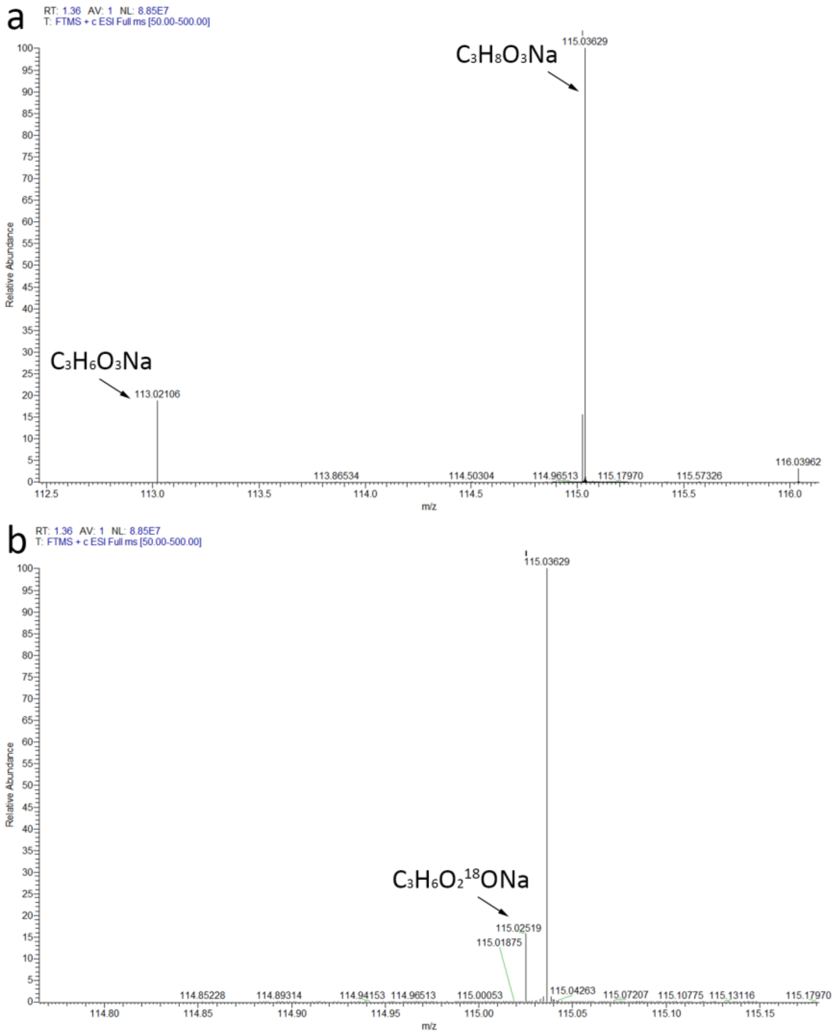

Supplementary Figure 17 | Liquid chromatography–mass spectrum of the photoelectrochemical glycerol oxidation products. a, LC-MS spectrum of the photoelectrochemical glycerol oxidation products obtained in isotope labeled electrolyte with H_2O containing 10% $H_2{}^{18}O$ (pH = 2). **b,** Enlarged version of (a).

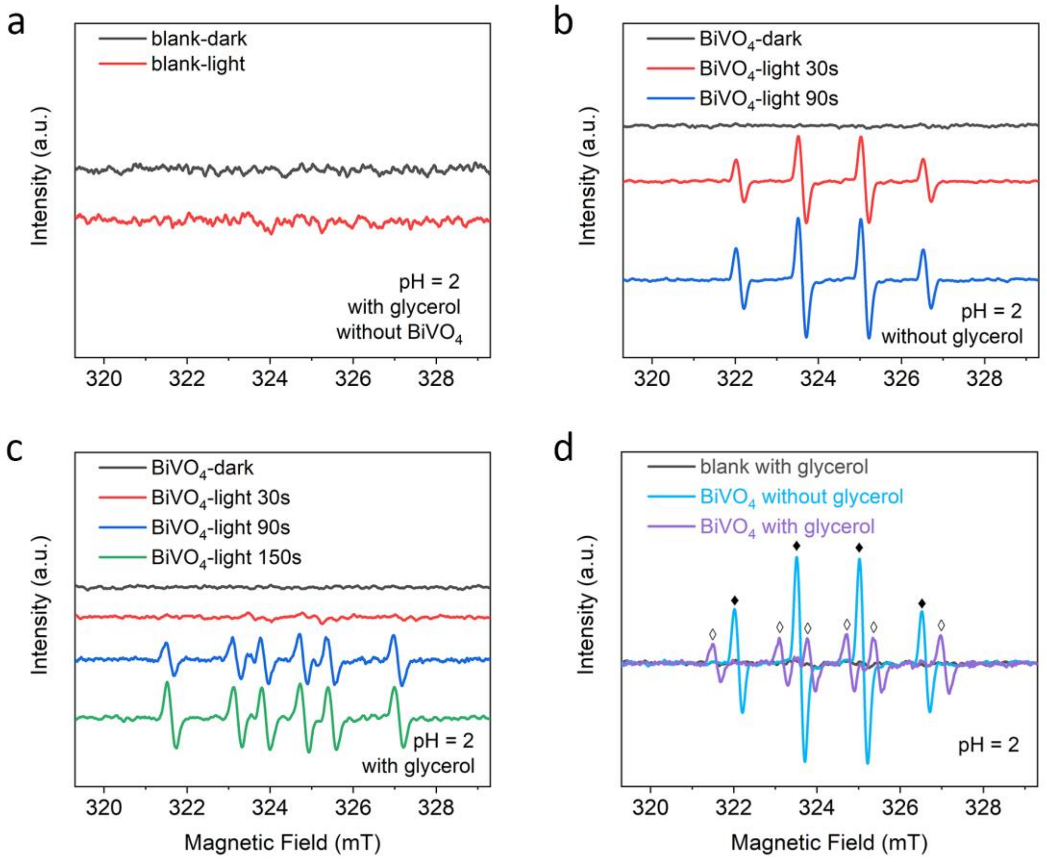

Supplementary Figure 18 | Room-temperature electron spin resonance spectra in 0.5 M Na_2SO_4 at pH = 2. a, ESR spectra with 0.1 M glycerol and without catalyst in solution. **b,** ESR spectra of $BiVO_4$ photocatalysts without glycerol. **c,** ESR spectra of $BiVO_4$ photocatalysts with 0.1 M glycerol. **d,** ESR spectra of three samples after 90 s illumination. The ones with "♦" belong to the spin adduct attributable to hydroxyl radicals. The ones with "◊" are attributed to the DMPO-glycerol radical adduct. Source data are provided as a Source Data file.

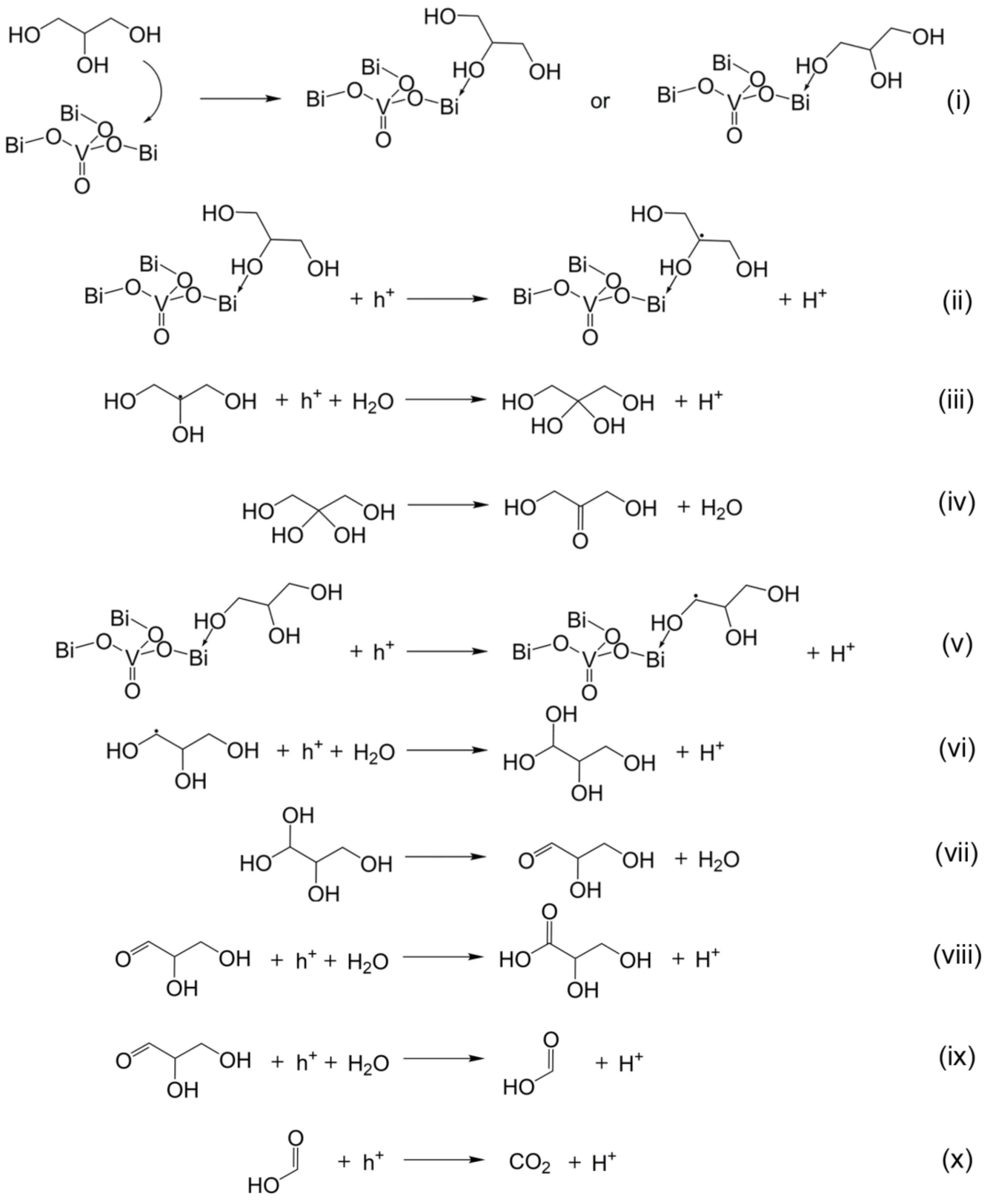

Supplementary Figure 19 | Photoelectrochemical glycerol oxidation pathways by $BiVO_4$.

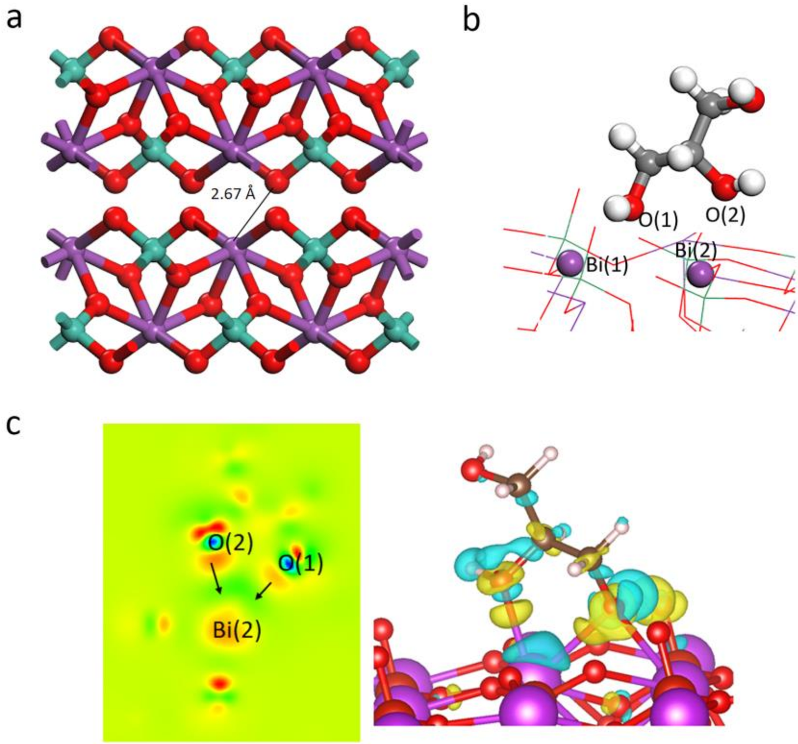

Supplementary Figure 20 | Structure model and charge density distribution of glycerol on $BiVO_4$. a, Structural model of bulk $BiVO_4$. **b,** Glycerol adsorbed on $BiVO_4$. **c,** 2D and 3D graphs of charge density difference: (gly/$BiVO_4$) – (gly) – ($BiVO_4$). Yellow is where charge density decreases and cyan is where charge density increases. Charge density on O 2p orbital decreases with charge transfer from O 2p to Bi.

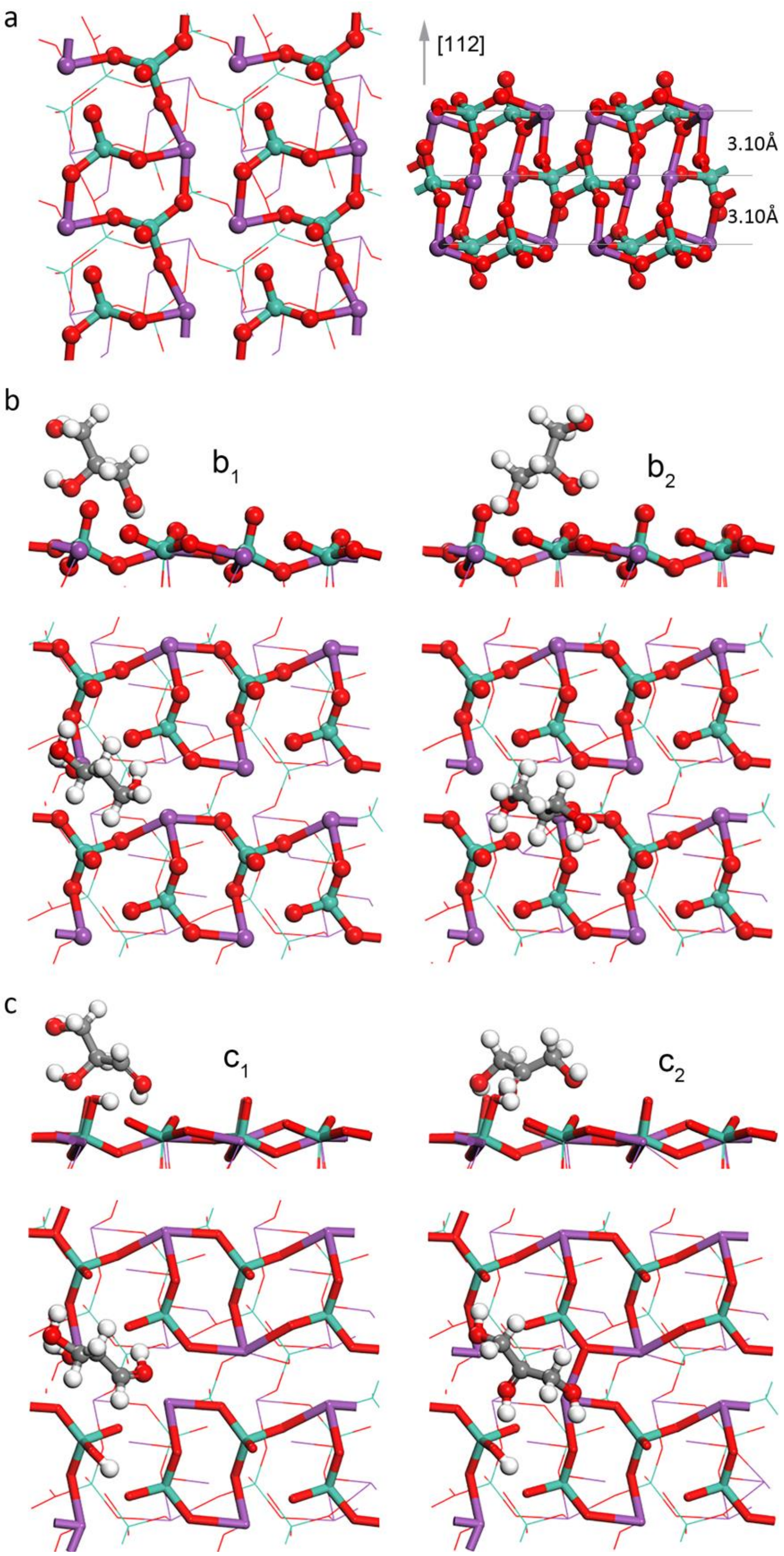

Supplementary Figure 21 | Structure model of $BiVO_4$(112) and glycerol oxidation reaction on $BiVO_4$(112). a, Structural model of $BiVO_4$(112) (top and side view). **b,** Adsorption of glycerol (b_1: terminal, b_2: middle) on $BiVO_4$(112). **c,** Oxidation of glycerol to carbon radicals (c_1: terminal, c_2: middle) on $BiVO_4$.

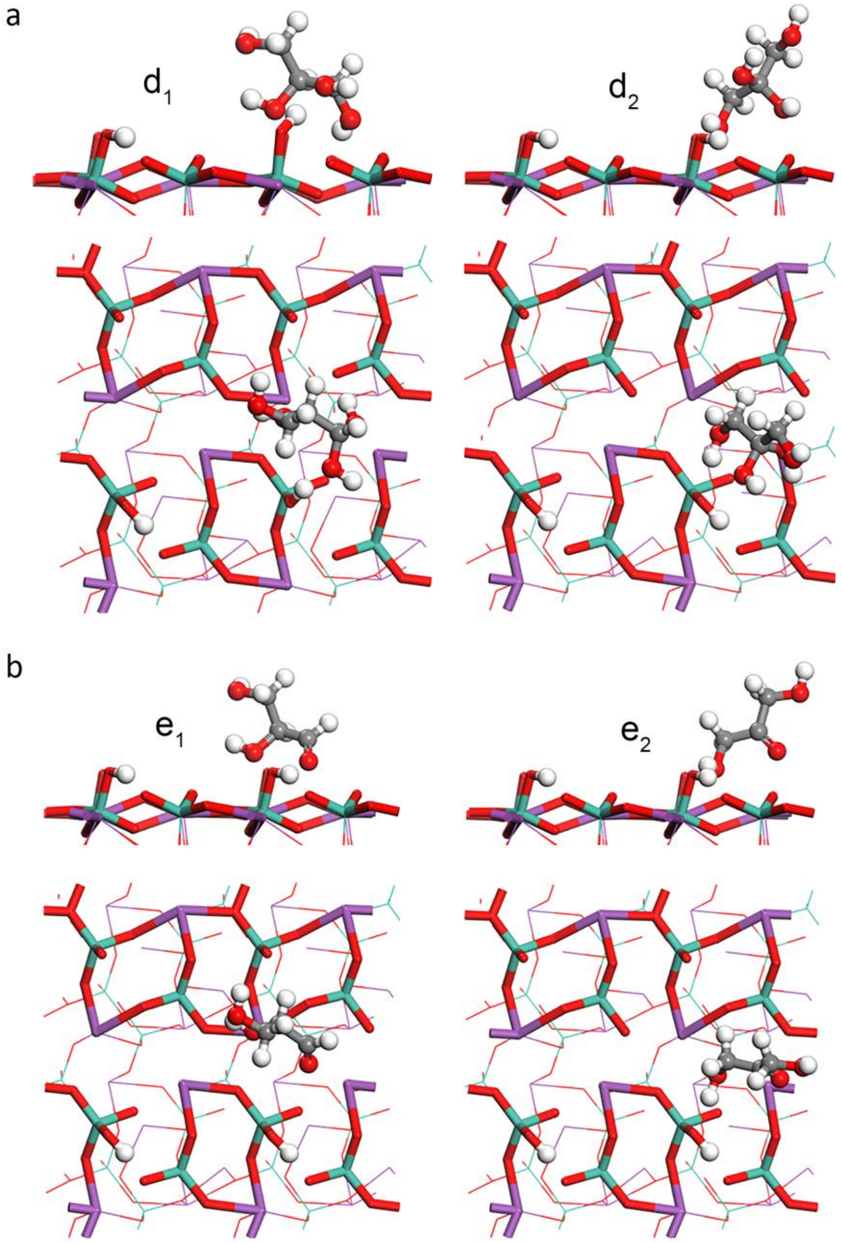

Supplementary Figure 22 | Structure model of glycerol oxidation reaction on $BiVO_4$(112). a, Reaction of carbon radicals with water to form gem-diol intermediates (d_1: terminal, d_2: middle) on $BiVO_4$(112). **b,** Dehydration of gem-diol and formation of products (e_1: terminal, e_2: middle) on $BiVO_4$(112).

Supplementary Table 1 | Production and carbon balance of glycerol oxidation reaction. Consumption rate of glycerol, production rate of glycerol derivatives, carbon balance and charge-to-chemical balance at pH = 2 and 1.2 V vs. RHE under AM 1.5G, 100 mW cm^{-2} illumination.

	**Consumption rate / mmol h^{-1} m^{-2}**	**Production rate / mmol h^{-1} m^{-2}**	**Conversion selectivity / %**	**Carbon balance / %**	**Charge-to-chemical balance / %**
Glycerol	307.8				
DHA		195.7	63.6		
Glyceric acid		30.7	10.0		
Formic acid		151.6	16.4		
Glycolic acid		0.9	0.2		
CO_2		7.9	0.9		
CO		2.0	0.2		
H_2O_2		141.8			
O_2		10.2			
Total				90.21	91.3

Supplementary Table 2 | Na^+ ESI source liquid chromatography–mass spectrum results acquired in pH = 2 isotope labeled electrolyte with H_2O containing 10% $H_2{}^{18}O$.

m/z	Intensity	Relative	Theo. Mass	Delta (mmu)	Composition
113.0211	166211.6	100	113.0209	0.14	$C_3H_6O_3Na$
115.0252	137668.2	100	115.0252	0.03	$C_3H_6O_2{}^{18}ONa$

Supplementary Table 3 | Photocatalytic performance of glycerol oxidation by $BiVO_4$ photocatalysts. Obtained in 0.5 M Na_2SO_4 at pH = 2 in a single cell under AM 1.5G, 100 mW cm^{-2} illumination with hole scavenger or hydroxyl radical scavenger.

**Production rate / mmol h^{-1} g^{-1}**	**DHA**	**Formic acid**	**Glyceric acid**	**DHA selectivity / %**
$BiVO_4$ photocatalysts	0.138	0.144	0.072	39.0
+ 0.1 M ammonia formate	0	--	0.139	0
+ 0.1 M *t*-butanol	0.143	0.227	0.066	32.7

Supplementary Table 4 | Catalytic performance of glycerol oxidation by hydroxyl radicals. Obtained in 0.5 M Na_2SO_4 at pH = 2 with or without $BiVO_4$ photocatalysts under dark or AM 1.5G, 100 mW cm^{-2} illumination.

**Production rate / μM h^{-1}**	**DHA**	**Formic acid**	**Glyceric acid**	**DHA selectivity / %**
Dark	494	2763	254	14.0
Light	1127	6840	570	13.2
Dark with $BiVO_4$ photocatalyst	508	2908	309	13.6
Light with $BiVO_4$ photocatalyst	1681	9623	698	13.9

Supplementary Note 1 | Mott-Schottky plots of the $BiVO_4$ photoanode.

The Mott-Schottky plot measured without glycerol under AM 1.5G illumination in 0.5 M Na_2SO_4 at pH = 2 in Supplementary Fig. 6 shows a clear shift towards lower potentials as compared to that measured in dark, indicating that surface states do play a role in water oxidation via a surface-state charging process.[S1] After adding glycerol in the PEC system, surface-state charging, *i.e.* hole accumulation, is largely suppressed. Supplementary Fig. 6b shows that Mott-Schottky plots do not change with and without light illumination in the presence of glycerol.

Supplementary Note 2 | ATR-FTIR spectra of $BiVO_4$ photoanode.

ATR-FTIR reflectance spectra of $BiVO_4$ photoanode treated with solution at pH = 2, 5 and 7 are shown in Supplementary Fig. 7. Vibration mode of sulfate anion of Na_2SO_4 has characteristic bands at about 1134 cm^{-1},[S2] 995 cm^{-1} and 1105 cm^{-1}.[S3] The symmetric and antisymmetric stretching vibration modes of C-O bond in primary alcoholic group and secondary alcoholic group of glycerol are at about 994 cm^{-1}, 1043 cm^{-1} and 1110 ~ 1120 cm^{-1}, respectively.[S4-S7] When pH decreases, the C-O bond symmetric and antisymmetric stretching peaks of glycerol increase distinctly, indicating that glycerol can better adsorb on $BiVO_4$ at lower pH. At the same time, the C-O bond vibration peak of the secondary alcoholic group shifts from 1109 cm^{-1} to 1118 cm^{-1} when pH decreases to 2, while the C-O bond vibration peaks of primary alcoholic group just shift by about 1 cm^{-1}. These indicate that C-O bond vibration frequency of the secondary alcoholic group is enhanced distinctly when pH decreases, which can be attributed to the analogous inductive effect by the enhanced electrostatic attraction between middle hydroxyl group and $BiVO_4$.[S7] H_2SO_4 has characteristic peaks at about 1140 cm^{-1} and 1160 cm^{-1}, assignable to the SOH symmetric and antisymmetric bending mode, respectively.[S8]

Supplementary Note 3 | Electrochemical impedance of $BiVO_4$ photoanode.

As shown in Supplementary Fig. 8c, enhanced adsorption of glycerol on $BiVO_4$ at lower pH together with higher productivity but poor desorption of oxidation products would lead to increased R_{total} with increasing potential or decreasing pH.

Supplementary Note 4 | Comparison of $BiVO_4$ porous nanoarrays and film photoanodes.

$BiVO_4$ film photoanode was prepared by the reported method.[S9] As shown in Fig. 1a and Supplementary Fig. 15, photocurrent density of $BiVO_4$ film photoanode is higher than that of porous $BiVO_4$ nanoarray photoanode for water oxidation. After adding glycerol, the photocurrent density of film photoanode is lower than that of porous nanoarray photoanode.

Supplementary Note 5 | Photoelectrochemical characterization of TiO_2 and Fe_2O_3 photoanode.

TiO_2 nanowires and Fe_2O_3 nanoporous photoanode were fabricated by hydrothermal methods.[S10, S11] TiO_2 nanowires shows excellent stability in pH = 2 electrolyte, and the photocurrent density increases a little after adding glycerol. As shown in Supplementary Fig. 16, no DHA is detected after PEC glycerol oxidation by TiO_2 nanowires. The liquid products efficiency is quite low for TiO_2 nanowires photoanode, which may because of competitive water oxidation and further oxidation of glycerol to CO_2. Fe_2O_3 nanoporous photoanode shows poor stability in pH = 2 electrolyte, and the photocurrent density increases a little after adding glycerol. Little DHA and other liquid products are detected after PEC glycerol oxidation by Fe_2O_3 nanoporous photoanode. The production rate, selectivity and efficiency of DHA and high-value added products obtained by Fe_2O_3 nanoporous photoanode are much less than that of $BiVO_4$ nanoporous photoanode.

Supplementary Note 6 | Liquid chromatography–mass spectrum results.

LC-MS results shown in Supplementary Fig. 17 and Table 2 indicate that ^{18}O originated from water in the electrolyte could be detected in the product - DHA after PEC glycerol oxidation reaction. This qualitative analysis suggests that water participates in the oxidation of hydroxyl group in glycerol.

Supplementary Note 7 | Photocatalytic performance of glycerol oxidation by $BiVO_4$ photocatalysts.

$BiVO_4$ scraped from the $BiVO_4$ nanoarrays were used as the photocatalysts. Hole scavenger (ammonium formate) and hydroxyl radical scavenger (*t*-butanol) were added in the photocatalysis system. The catalytic performance (Supplementary Table 3) shows that after adding hole scavenger, DHA could not be produced, and meanwhile, after adding hydroxyl radical scavenger, the production of DHA was not affected too much. It indicates that holes should directly participate in the glycerol oxidation reaction.

Supplementary Note 8 | Catalytic performance of glycerol oxidation by hydroxyl radicals.

Hydroxyl radicals were introduced in this glycerol photocatalysis system by Fenton reaction.[S12] 0.3 M H_2O_2 and 3 mM Fe_2SO_4 were added into 0.5 M Na_2SO_4 at pH = 2 with 0.1 M glycerol under stirring in a single cell. As shown in Supplementary Table 4, the selectivity of DHA by oxidation of OH radicals is as low as 13~14 %. The results indicate that this homogeneous glycerol oxidation process driven by hydroxyl radicals could only produce DHA with low selectivity. On the other hand, it also shows that it is harder for middle hydroxyls of glycerol to be oxidized than terminal hydroxyls.

Supplementary Note 9 | Detection of glycerol oxidation reaction intermediates.

Room-temperature Electron Spin Resonance (ESR) spectra were collected to explore the reaction intermediates. $BiVO_4$ scraped from the $BiVO_4$ nanoarrays were used as the photocatalysts. The ESR spectra in Supplementary Fig. 18 show that without $BiVO_4$, glycerol could not be activated into radicals. Without glycerol, $BiVO_4$ photocatalysts could oxidize water into hydroxyl radicals. After adding glycerol in the system, instead of hydroxyl radicals, glycerol radicals were detected, indicating that glycerol could be stimulated into radicals by $BiVO_4$ photocatalysts. The ESR spectrum of DMPO-glycerol radical adduct observed here is similar to that of the DMPO-alcohol radical adducts.[S13]

Supplementary Note 10 | Photoelectrochemical glycerol oxidation pathways on $BiVO_4$.

As shown in Supplementary Fig. 19, we infer that the oxidation of glycerol to DHA could occur via the following steps: (i) Adsorption of glycerol on $BiVO_4$ photoanode. The terminal or middle hydroxyl group of glycerol can adsorb on bismuth of $BiVO_4$. (ii) Photogenerated holes would transfer to the middle carbon of glycerol and oxidize the middle carbon into carbon radical. (iii) Carbon radicals would further react with holes and water to form middle gem-diol intermediates. (iv) The dehydration of gem-diol would occur immediately and DHA could be produced. (v-x) The oxidation of terminal hydroxyl group.

Supplementary Note 11 | Structural parameters of glycerol on $BiVO_4$.

Interlaminar distance of Bi-O is 2.67 Å (Supplementary Fig. 20).

L(O1-Bi1) = 2.96 Å L(O1-Bi2) = 2.81 Å L(O2-Bi1) = 2.63 Å

The distance and strength of Bi-O between layers of $BiVO_4$ and O(glycerol)-Bi($BiVO_4$) are similar. There exists electrostatic attraction between Bi^{3+} and O^{2-}.

Bader charges:

O(1): -1.73 |e| O(2): -1.68 |e|

Bi(1): +2.98 |e| Bi(2): +2.78 |e|

Supplementary References

1. Klahr, B., Gimenez, S., Fabregat-Santiago, F., Hamann, T. & Bisquert, J. Water oxidation at hematite photoelectrodes: The role of surface states. *J. Am. Chem. Soc.* **134**, 4294-4302 (2012).
2. Nakamura, M., Ikemiya, N., Iwasaki, A., Suzuki, Y. & Ito, M. Surface structures at the initial stages in passive film formation on Ni(111) electrodes in acidic electrolytes. *J. Electroanal. Chem.* **566**, 385-391 (2004).
3. Tong, H. J., Reid, J. P., Dong, J. L. & Zhang, Y. H. Observation of the crystallization and supersaturation of mixed component $NaNO_3$-Na_2SO_4 droplets by FTIR-ATR and Raman spectroscopy. *J. Phys. Chem. A* **114**, 12237-12243 (2010).
4. The spectral database system for organic compounds at the National Institute of Materials and Chemical Research in Japan is available at http://sdbs.db.aist.go.jp/sdbs/cgi-bin/cre_index.cgi.
5. Schnaidt, J., Heinen, M., Denot, D., Jusys, Z. & Behm, R. J. Electrooxidation of glycerol studied by combined in situ IR spectroscopy and online mass spectrometry under continuous flow conditions. *J. Electroanal. Chem.* **661**, 250-264 (2011).
6. Copeland, J. R., Santillan, I. A., Schimming, S. M., Ewbank, J. L. & Sievers, C. Surface interactions of glycerol with acidic and basic metal oxides. *J. Phys. Chem. C* **117**, 21413-21425 (2013).
7. Larkin, P. *Infrared and Raman spectroscopy : principles and spectral interpretation*. Elsevier (2011).
8. Hintze, P. E., Kjaergaard, H. G., Vaida, V. & Burkholder, J. B. Vibrational and electronic spectroscopy of sulfuric acid vapor. *J. Phys. Chem. A* **107**, 1112-1118 (2003).
9. Wang, S. *et al.* New $BiVO_4$ dual photoanodes with enriched oxygen vacancies for efficient solar-driven water splitting. *Adv. Mater.* **30**, 1800486 (2018).
10. Liu, B. & Aydil, E. S. Growth of oriented single-crystalline rutile TiO_2 nanorods on transparent conducting substrates for dye-sensitized solar cells. *J. Am. Chem. Soc.* **131**, 3985-3990 (2009).
11. Zhang, P., Wang, T., Chang, X., Zhang, L. & Gong, J. Synergistic cocatalytic effect of carbon nanodots and Co_3O_4 nanoclusters for the photoelectrochemical water oxidation on hematite. *Angew. Chem. Int. Ed.* **55**, 5851-5855 (2016).
12. Zepp, R. G., Faust, B. C. & Hoigne, J. Hydroxyl radical formation in aqueous reactions (pH 3-8) of Iron(II) with hydrogen-peroxide: the photo-Fenton reaction. *Environ. Sci. Technol.* **26**, 313-319 (1992).
13. Wu, W. *et al.* A new insight into the photocatalytic reduction of 4-nitroaniline to p-phenylenediamine in the presence of alcohols. *Appl. Catal. B Environ.* **130**, 163-167 (2013).
